# Applications of social marketing for implementation science: a scoping review

**DOI:** 10.1186/s13012-025-01458-z

**Published:** 2025-11-29

**Authors:** Heather Colquhoun, Moriah E. Ellen, Jamie Brehaut, Nedra Kline Weinreich, Coby Morvinski, Sareh Zarshenas, Tram Nguyen, Justin Presseau, Nicola McCleary, Heather A. Shepherd, Armaghan Dabbagh, Enola Proctor

**Affiliations:** 1 Department of Occupational Science and Occupational Therapy, University of Toronto, 160-500 University Ave, Toronto, ON M5G 1V7 Canada; 2 Department of Health Policy and Management, Guilford Glazer Faculty of Business and Management and Faculty of Health Sciences, Ben-Gurion University of the Negev, Beer-Sheva, Israel; 3 Methodological and Implementation Research, Ottawa Hospital Research Institute, Ottawa, ON Canada; 4 School of Epidemiology and Public Health, University of Ottawa, Ottawa, ON Canada; 5 Weinreich Communications Ltd, Jerusalem, Israel; 6 Department of Management, Guilford Glazer Faculty of Business and Management, Ben-Gurion University of the Negev, Beer-Sheva, Israel; 7 Institute of Health Policy, Management and Evaluation, Dalla Lana School of Public Health, University of Toronto, Toronto, Canada; 8 Brown School, Washington University in St. Louis, St. Louis, MO USA

**Keywords:** Social Marketing, Knowledge translation, Implementation science, Intervention

## Abstract

**Background:**

Implementation science has a history of drawing from other fields to advance its science, yet understanding how approaches from marketing might enhance the field remains a largely untapped area of theoretical and methodological potential. Social marketing (i.e., applying commercial marketing to solve social or health problems) is a branch of marketing that shares many conceptual features with implementation science (e.g., behaviour change), but remains an unrealized opportunity for synergy. This review aimed to 1) describe studies that have tested social marketing interventions in controlled designs; 2) describe these interventions including their context, mechanism, and outcome; and 3) propose social marketing approaches that might be usefully applied to implementation science.

**Methods:**

This scoping review, with a team consensus discussion, followed JBI (formerly the Joanna Briggs Institute) methodological guidance and included a team of researchers and practitioners in implementation, marketing, and social marketing. Twelve databases were searched. Studies were included that 1) utilized a randomized or non-randomized controlled intervention design; and 2) tested a social marketing intervention as defined by five essential social marketing criteria. Two reviewers independently completed all screening and extraction. Variables extracted included intervention details per social marketing criteria and the intervention’s context, mechanism, and outcome. Team consensus discussions of the scoping review results were used to determine approaches that might be usefully applied more broadly across implementation science.

**Results:**

Screening of 4,867 citations yielded 28 included studies published from 1999–2023. All topics were from the health field and included nutrition (13, 46%), sexual health/family planning (6, 21%), physical activity (3, 11%), child safety (1, 4%), cancer screening (1, 4%), fall prevention (1, 4%), worksite safety (1, 4%), sanitation (1, 4%), and substance abuse (1, 4%). Novel theories identified included ‘Exchange Theory’ and ‘Consumer Information Processing Model’. Proposed approaches to consider for application included: leverage emotions; design for appeal; consider what your audience values; understand the price; understand the place; emphasize competitive advantage; and use branding.

**Conclusions:**

This review examined the application of social marketing theories and approaches to implementation science. Applying social marketing approaches could invigorate novel and creative thinking in implementation science.

**Registration:**

Open Science Framework Registration link: osf.io/6q834.

**Supplementary Information:**

The online version contains supplementary material available at 10.1186/s13012-025-01458-z.

Contributions to the literature

- Social marketing is a field that has applications to implementation science.
- Social marketing and implementation science have clear overlap but also differ in perspectives and implications.
- Seven approaches used in social marketing could enhance implementation efforts: emotion; appeal; value; price; place; competition; and branding.
- Future work should systematically design and test social marketing approaches in implementation efforts.

## Background

Proctor and colleagues’ 2021 *Implementation Science* article challenged the field of implementation science to pay greater attention to market forces, market demands, and market relevant concepts [1]. They proposed that asking, “if we were to build this, would they buy?—and if so, at what price and in what quantities?” could catalyze novel and potentially beneficial approaches to behaviour change in a health delivery context [1] (p. 2). The authors suggest these two different, but complementary, fields (i.e., marketing and implementation science) share some priorities, values, and norms. Efforts to foster communication, shared language, and collaboration between implementation science and marketing are needed.

Marketing science has aims consistent with implementation science (e.g., disseminating knowledge, changing behaviour, persuading others), yet implementation scientists have rarely considered literature, evidence, or supporting frameworks from marketing science. One relevant sub-area of marketing science is social marketing [2]. Social Marketing is “the application of commercial marketing techniques to the analysis, planning, execution, and evaluation of programs designed to influence the voluntary behaviour of target audiences in order to improve their personal welfare and that of society” [2](p. 7). Initially conceptualized in 1971 by Kotler and Zaltman, social marketing is a dynamic and interdisciplinary field that has evolved to draw insights from various disciplines such as psychology, sociology, anthropology, economics, and communications theory [3]. The social marketing approach aims to comprehensively understand and leverage these diverse fields to effectively influence and shape people's behaviours for the benefit of individuals or society overall. Social marketing extends its reach across a broad spectrum of societal concerns and issues, including diverse subjects that impact human well-being and the community at large (e.g., smoking cessation, nutrition, vaccination, domestic violence, tourism, recycling, action on climate change, and voter engagement) [4–6]. Social marketing is defined by one essential principle: social value creation through the exchange of social offerings; and four essential concepts: social behavioural influence; citizen/customer/civic society orientation focus; social offerings; and relationship building [7].

As a central concept, social marketing adapts a strategic approach from commercial marketing called the “marketing mix,” or the 4 Ps: Product; Price; Place; and Promotion [8, 9]. Product (i.e., what is being offered) in social marketing is most often the behaviour you want the intended audience to adopt, along with the benefits associated with the desired behaviour. Price (i.e., the amount that consumers will be willing to pay for a product) in social marketing is what the intended audience has to give up to adopt the behaviour. Price could be monetary, but it also may include intangible costs such as time and effort, or changing old habits. Place (i.e., where and how a product is available to customers) in social marketing is where, when, and how the intended audience can be reached and access the behaviour… Promotion (i.e., communicating the product/offering to the target audience) in social marketing is how the intended audience becomes aware of the behaviour being encouraged and the associated benefits. Once these marketing concepts are re-defined into social marketing concepts, the alignment to implementation science becomes clearer (e.g., the intended behaviour change, the changing of habits, where are people when they have the opportunity to change their behaviour, how can the benefits of behaviour change be promoted).

We are aware of two studies that specifically addressed social marketing in implementation science. One involved the use of audience segmentation (i.e., design specifically to the needs and wants of the various target groups within your audience) to design an implementation strategy [10]The second was a review by Davies and colleagues that examined the use of theory in implementation research [11]. Davies’ review found eight studies (of the 53 included) that applied social marketing theory in what they termed as ‘conceptual use’ (as opposed to explicit use). Although these studies were deemed by the authors to have invoked social marketing theory generally through the use of academic detailing and outreach visits, none of them specified a social marketing theory.

As a first step to determining the social marketing approaches that show promise to be applied in implementation science, a detailed summary of tested social marketing interventions combined with a discussion of these interventions by those with experience in implementation science and social marketing could yield useful applications. Thus, our specific objectives were to:

1. Describe the extent, range, and nature of studies that have tested social marketing interventions in controlled designs;
2. Describe the social marketing interventions using five essential social marketing criteria [7] and their context, mechanism, outcome (CMO) [12]; and
3. Propose social marketing approaches that might be usefully applied to implementation science (team consensus activity).

## Methods

Our scoping review was conducted using the JBI Methodological Guidance for Scoping Reviews [13], including the publication of our scoping review protocol outlining the methodological details [14]. We reported the review according to the Preferred Reporting Items for Systematic Reviews and Meta-Analyses Extension for Scoping Reviews (PRISMA- ScR) [15]. Our principal search strategy was reviewed using the Peer-Review of Electronic Search Strategies (PRESS) criteria [16]. There were three deviations from our pre-registered and published protocol [14]. The first was to remove the extraction of five desirable, but not essential, social marketing techniques. We found intervention reporting was too limited to extract these additional techniques reliably from the papers we included, and instead put our efforts into a robust extraction of the five essential social marketing criteria. Second, we could not use our planned conceptual framework of context to organize the context variables. The context framework we had planned to use was specific to implementation science and was not aligned with the contextual features found in social marketing interventions. For example, concepts specific to implementation science, such as ‘internal organization processes,’ were not central to these social marketing interventions [17], which primarily focused on broader community-based contexts. Last, our methods for determining novel social marketing intervention approaches to integrate into implementation science (objective 3) were expanded (see detailed description in the analysis section of the methods).

### Eligibility criteria

As per our protocol, we included studies published from 1971 onwards that: 1) utilized a controlled intervention study; and 2) tested a social marketing intervention that adhered to the five essential social marketing criteria [7]. Our population was adults who received social marketing interventions. Our concept was social marketing as defined by French and Russell-Bennett [7]. This definition was recommended by our social marketing practitioner (NKW) as the most useful definition, and is organized into one essential principle (i.e., social value creation through the exchange of social offerings) and four essential concepts (i.e., social behavioural influence; citizen/customer/civic society orientation focus; social offerings; and relationship building). Although these five criteria and their associated definitions were a helpful starting point for our review, operationalizing them in a knowledge synthesis that relies on clarity of reporting was challenging. It became necessary for us to add further details to the definitions to reliably apply them to potential studies for inclusion. Further clarifications for the definitions were determined in consultation with the social marketing practitioner on our team (NKW). See Table 1 for a detailed description of the five essential social marketing criteria with additional points of clarity. We did not exclude by setting (e.g., tourism, climate change, health promotion). We chose 1971 as the first year of inclusion as it marks the birth of the field of social marketing with Kotler and Zaltman’s original article [3]. We included only English language studies due to feasibility and resources. We excluded designs other than controlled intervention studies, those that did not adhere to the essential criteria, commentaries, reviews, and conference abstracts. Also, we excluded studies employing social marketing interventions directed at anyone under 18 years of age, as implementation interventions are less often directed specifically at children or adolescents. We did include studies in which children or adolescents were beneficiaries of an intervention, but the intervention itself was directed at adults (e.g., improving childhood nutrition by targeting parents' behaviour).

**Table 1:** Social marketing criteria

**Criteria**	**Explanation **	**Additional specific criteria used to operationalize the explanation **
**Essential Principle**		
**1. Social value creation** **through the exchange of** **social offerings (ideas,** **products, service,** **experience, environments,** **systems)**	The aim and objectives of bringing about social value and improvement and/or the reduction of social problems through a reciprocal exchange of resources or assets at the individual, community, societal or global level. Social policy, strategy, understanding ideas, products, services and experiences are developed that will enable and assist citizens to derive social benefits individually and collectively.	A reduction of a social problem through a reciprocal exchange of social offerings (ideas, products, service, experience, environments, systems) at the individual, community, societal or global level.
**Essential concepts (** ***n*** **=4)**		
**1. Social behavioural** **influence**	Behavioural analysis is undertaken to gather details of what is influencing behavioural patterns and trends. Interventions are developed that seek to influence specific behaviours and clusters of related behaviours. Specific actionable and measurable behavioural objectives and indicators are established. A broad range of behavioural theory is used to analyze implement and evaluate interventions. These behaviours could be upstream, midstream or downstream.	Study describes information obtained (either through formative work or using other literature) about the reasons why the target group is not engaging in the behaviour. Theory may or may not be used, and may or may not be used explicitly.
**2. Citizen/customer/civic** **society orientation focus**	Policy planning, delivery and evaluation are focused on building understanding and interventions around citizen beliefs, attitudes behaviours, needs and wants. A range of different research analyses, combining qualitative and quantitative data gathering, is used and synthesized to plan, deliver and review interventions.	The 4 Ps (product, price, place, promotion) are either explicitly or implicitly described. If implicitly described, it is possible to extract specific information on the 4 Ps.
**3. Social offerings (idea,** **product, service experience)**	Target markets (citizens, policy-makers or any other relevant audience) are offered products, ideas, understanding, services, experiences, systems and environments that provide value and advantage. In most cases such social offerings are positive in nature, for example they provide protection or the promise of better health. However, these social offerings can also involve the imposition of restrictions on freedom such as speed limits on motorways that have collective support and benefit.	The target group is provided with a social offering of some sort (e.g., a product, education, an idea).
**4. Relationship building**	The establishment of collective responsibility and the collective right to wellbeing is developed through a process of engagement and exchange. Citizens, policy-makers or stakeholders are engaged in the selection of priorities, and the development, design, implementation and evaluation of interventions.	A process of engagement and/or exchange has occurred. This includes co-creation, engagement with community based organizations that support the target group, and formative work that was done specifically with those who are being studied.

### Sources of data, search strategies, and data collection

Our search strategy was designed by SZ and HC in consultation with a University of Toronto Librarian. We searched 12 databases, including Ovid MEDLINE, Scopus, Embase, Cochrane libraries, CINAHL, PsycINFO, ERIC, PubMed, the Arts and Humanities Citation, Social Science Citation, and Science Citation Indices (see Additional File 1 for all search strategies of the databases). In addition, we hand searched the reference lists of the four social marketing reviews we were aware of [18–21], key social marketing journals (e.g., Social Marketing Quarterly, Journal of Social Marketing), and the included articles.

### Data screening

After removing duplicates using EndNote, search results were downloaded into the Covidence review platform (https://www.covidence.org, Accessed May 2023). All screening (title/abstract and full text) was completed independently by two of four screeners (SZ; HC; HAS; AD).

### Data extraction

An a priori data extraction guide was created by SZ and HC, reviewed by the research team, and piloted to ensure clarity of variables. The following variables were extracted:

- Objective 1: Extent, range, and nature of studies: Year study conducted; countries/regions of origin; funded vs unfunded; journal name; overall topic (e.g., health); detailed topic; behaviour being changed; distinct steps to how the intervention was designed; theory used; strategies to promote implementation; sample size; control group; results.
- Objective 2: General social marketing intervention description: the five essential social marketing criteria; context, mechanism (i.e., described mechanism of change); and outcome in addition to behaviour (i.e., attitudes, knowledge, other). Companion publications (e.g., formative evaluations) were located if referenced in the included paper and used for extraction but not for the final count of included papers.
- Objective 3: Data were not extracted for objective three and instead emerged from team consensus discussions.

### Data analysis

Descriptive quantitative analysis was used for year, country, funding, journal, sample size, control group, theory/framework, overall and detailed topic, behaviour to be changed, and outcome. The intervention design steps were summarized by quantity of similar steps (e.g., how many studies used a focus group). HC completed this analysis, and it was verified by HAS. HC synthesized the categories of intervention strategies to promote implementation using conventional content analysis [22] based on the brief intervention description extracted. This inductive process involved assigning a category to each social marketing intervention component and grouping accordingly. This was completed in multiple rounds by two members of the research team (HC;HAS), first independently, followed by consensus discussions to arrive at a final category list. HC and HAS deductively coded mechanisms using a commonly applied mechanism framework for behaviour change, the Capability, Opportunity, Motivation – Behaviour (COM-B) framework [23]. For each study, the extracted mechanisms were coded as C, O, M or any combination, first independently, followed by consensus discussions. The extracted data on the five essential social marketing criteria and the context were too unique to the individual study to meaningfully synthesize, and are presented as raw data in Additional File 2. The results of the papers were not synthesized as is appropriate for a scoping review that did not undertake any risk of bias assessment.

### Team consensus discussion methods

To analyze and interpret the data for the third objective (i.e., propose social marketing approaches to apply to implementation science), we undertook the process described in our protocol [14] with some enhancements. Each research team member read the full table of all extracted information for all studies prior to the meeting. The aim was to ensure the team was thoroughly familiar with all data collected, including details of the social marketing interventions. The team meeting was held for 2.5 h and included a 10-min presentation by HC on implementation science (to frame the discussion for the marketing/social marketing team members), a 10-min presentation by NKW on social marketing (to frame the discussion for the implementation scientists on the team), and a review of the data from the scoping review. The presentation on implementation science included a review of advancements in the field over the last 15–20 years that involved input from the entire team. The discussion was kept open with the aim of achieving consensus but was framed by the following guiding questions: What is similar between the two fields; what is different; what insights general or specific ideas could implementation science borrow from social marketing. In this discussion, the team was asked to consider the presentations delivered at the start of the meeting and the extraction file of the included studies. The meeting was audio recorded and two team members served as note takers (HAS;AD). The meeting discussion, recordings and notes were summarized into potential approaches including definitions and examples by HC, ME and NKW and confirmed by the entire research team in three separate opportunities for editing, feedback, and consensus. The experience and seniority of the team involved in these discussions were as follows: Two senior implementation science researchers (JB;EP), three mid-career implementation science researchers (HC;ME;JP), one mid-career marketing researcher (CM); four junior/trainee implementation science researchers (TN;NM;HAS;AD); one research associate (SZ); and one senior social marketing practitioner (NKW).

## Results

**Fig. 1:**
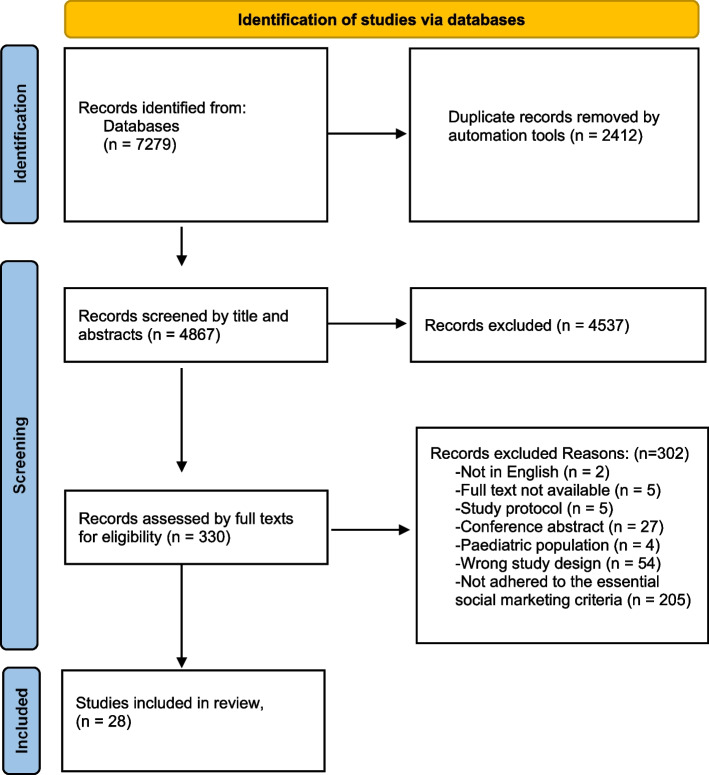
PRISMA flow diagram

We screened 4,867 titles and abstracts. After excluding 4,537 of these studies, we completed a full text review of 330 studies. Common reasons for exclusion at full text were not adhering to the essential social marketing criteria (*n* = 205) and excluded for study design (*n* = 54). Twenty-eight studies were included in the review (see Fig. 1 for the PRISMA-ScR chart). The search is current to January 30th, 2024.

### Study characteristics

The earliest included study was published in 1999 (i.e., no studies were found between 1971 and 1998). Years of publication since that time were as follows: 2000–2004 (*n* = 3), 2005–2009 (*n* = 3), 2010–2014 (*n* = 5), 2015–2019 (*n* = 10), and 2020–2023 (*n* = 6). Across the 28 studies, 16 different countries were represented. The studies were conducted in the United States (*n* = 11, 39%), two each in India and Iran, and one in each of the remaining 13 countries (i.e., Australia, Bangladesh, China, Ecuador, Japan, Netherlands, New Zealand, Nigeria, Slovenia, Tanzania, Thailand, Uganda, United Kingdom). Twenty-five (89%) studies reported at least one funding source. There were 25 different journals represented across the 28 included studies. Three journals were featured twice in the list (American Journal of Preventive Medicine, Bulletin of the World Health Organization, and Public Health Nutrition). Twelve studies included < 500 participants. Three studies included 501–1000 participants. Five studies included 1001–5000 participants. Three studies included more than 5001 participants, with one of these studies including > 50,000 participants. Five studies reported a community (i.e., stores, parks, cafeterias) as a sample size. Of the 28 studies, 16 (57%) were randomized, and 12 (43%) were not randomized (i.e., had matched controls).

### Health topics addressed

All topics were related to health with the following detailed topics: Nutrition (13, 46%); Sexual Health/Family Planning (6, 21%); Physical Activity (3, 11%); Child Safety (1, 4%); Cancer Screening (1, 4%); Fall Prevention (1, 4%); Worksite Safety (1, 4%); Sanitation (1, 4%); and Substance Abuse (1, 4%). See Table 2 for a complete summary of the topics and the related behaviours to be changed.

**Table 2:** Specific topics and related specific behaviours

**Specific Topic**	**No (%),** ***n*** **=28**	**Specific Behaviours**
Nutrition	13 (46%)	• Increasing the purchase (and therefore the consumption) of fruit and vegetables • Decrease purchase of unhealthy (high sugar) items by customers and decreasing the selling of unhealthy items at the grocery store. • Parents introducing eggs into the diets of infants from age 6-9 months. • Increase use of vitamins by women of child bearing age. • Improve health through lifestyle changes • Increase fruit and vegetable intake and frequency of family meals • Behaviours related to healthy eating (children's dietary intake, parental skills at healthy cooking). • Increase Fruit Intake Among Community College Students • Increase purchase of healthier foods. • The purchase and use of Iron-fortified Soy Sauce among Women in China • Making healthier food choices • Substitute traditional unfortified cooking oil with vitamin A-fortified sunflower oil in household cooking •Husband engagement re nutrition practices during their wives’ pregnancies
Sexual Health/Family Planning	6 (21%)	• Condom use by sex workers • Condom use by female sex workers • Use of contraception • Uptake of pap smear • HIV risk behaviours (unprotected sex, sexual communication with partner) • Engage in 12 preconception behaviours
Physical Activity	3 (11%)	• Increasing physical activity in parks • Increase physical activity in middle-aged and elderly people (40-79 yrs) - promoting aerobic PA, flexibility and muscle strengthening activities. • Increasing walking and physical activity
Child Safety	1 (4%)	• Parents installing booster seats for their children in cars
Cancer Screening	1 (4%)	• Increase breast and cervical cancer screening among Muslim women in New York City
Fall Prevention	1 (4%)	• Older adults attending balance classes for fall prevention
Worksite Safety	1 (4%)	• Use personal protective equipment in constructing subway stations
Sanitation	1 (4%)	• Uptake of individual latrines in households.
Substance Abuse	1 (4%)	• Decrease retailer selling of underage alcohol, thus decreasing alcohol consumption of those less than 18.

### Theories/frameworks used

Seventeen different theories or frameworks were used across the 28 studies. The most common, Health Belief Model [24], was used five times (18%). In total, 23/28 (82%) of the studies used at least one of these 17 theories or frameworks. Eleven studies (39%) reported using what we termed general approaches or knowledge areas, areas that while they may explain behaviour to some degree, were not labelled as theories or frameworks. These included a ‘Social marketing theory/approach’, ‘Theory of Nudging’, ‘Consumer Behaviour Theory’, and ‘Health Psychology Theory’. No theories or knowledge areas were reported in seven studies (25%). Table 3 provides a complete summary of all theories and frameworks.

**Table 3:** Theories Used

**Theory/Framework**	**Number of Studies (%),** ***n*** **=28**
Health Belief Model [24]	5 (18%)
Exchange Theory [25]	2 (7%)
Transtheoretical Model (Stages of Change) [26]	2 (7%)
Community-based Participatory Methods [27]	1 (4%)
Consumer-based Health Communication Model [28]	1 (4%)
Consumer Information Processing Model [29]	1 (4%)
He Pikinga Waiora Framework (Cultural-specific framework) [30]	1 (4%)
Diffusion of Innovations [31]	1 (4%)
Ecological Model [32]	1 (4%)
RE-AIM [33]	1 (4%)
Sexual Health Model [34]	1 (4%)
Social Cognitive Theory [35]	1 (4%)
Socioecological Framework [36]	1 (4%)
TARPARE Model [37]	1 (4%)
Theoretical Domains Framework [38]	1 (4%)
Theory of Planned Behaviour [39]	1 (4%)
Theory of Reasoned Action [40]	1 (4%)
General, unspecified: ‘Social marketing theory/approach (*n*=8)’, ‘Theory of Nudging (*n*=1)’, ‘Consumer Behaviour Theory (*n*=1)’, ‘Health Psychology Theory’(*n*=1)	11 (39%)
None reported	7 (25%)

TARPARE: T: The Total number of persons in the segment; AR: The proportion of At Risk persons in the segment; P: The Persuadability of the target audience; A: The Accessibility of the target audience; R: Resources required to meet the needs of the target audience; and E: Equity, social justice considerations

*RE-AIM* Reach, Effectiveness, Adoption, Implementation, and Maintenance

### Steps for intervention design

As an essential criterion for social marketing, all studies used formative work as a step for intervention design. Although not all studies specifically stated the steps they used to design their interventions, we were able to extract a series of steps to intervention design in 25/28 (89%) of the studies. The most common steps to formative work included focus groups (13/25, 52%) and interviews (9/25, 36%). Seven studies (7/25, 28%) explicitly stated they applied a theory or a framework to their intervention design. Other studies described co-design/co-creation (2/25, 8%), designing specific to the four Ps (5, 20%), piloting a draft version of the intervention (2/25, 8%), and using a communications agency to design the materials used in the intervention (1, 4%). See Table 4 for a complete listing of the various approaches to intervention design.

**Table 4:** Intervention design steps

**First author, year**	**Design Steps **
Aitken, 2013 [41]	1. Behavioural analysis from the literature; 2. Application of theory, and/or frameworks
Ayala, 2013 [42]	1. Behavioural analysis from the literature; 2. Interviews
Basu, 2004 [43]	Not described
Brimblecombe, 2020 [44]	1. Co-design strategy
Cohen, 2013 [45]	1. Survey
DiGuiseppi, 2014 [46]	1. Interviews; 2. Focus groups; 3. Observations
Ford, 1999 [47]	1. Stakeholder discussion
Iannotti, 2017 [48]	1. Identify audience; 2 Consider levels of influence (individual, community); 3. Design using the four Ps
Kamada, 2018 [49]	1. Environmental scan; 2. SWOT (Strengths, Weaknesses, Opportunities, Threats) analysis; 3. Application of theory, and/or frameworks; 4. Design using the four Ps; 5. Pretest materials.
Kamin, 2016 [50]	1. Develop pilot intervention with participants; 2. Feedback from participants; 3. Adapt intervention
Lawrence, 2003 [51]	1. Define preliminary intervention components; 2. Focus groups;
Lutalo, 2010 [52]	Not described.
Maddison, 2019 [53]	1. Review similar intervention; 2. Review related guidelines; 3. Focus groups
Tietyen Mullins, 2020 [54]	1. Review related literature; 2. Focus group; 3. Design using the four Ps
Nguyen, 2018 [55]	Not described.
Olubodun, 2022 [56]	1. Focus groups; 2. Survey
Pattanayak, 2009 [57]	1. Interviews; 2. Focus groups; 3. Survey
Robinson, 2002 [58]	1. Focus groups; 2. Community input to ensure cultural alignment
Seguin-Fowler, 2021 [59]	1. Interviews; 2. Observations; 3. Application of theory, and/or frameworks; 4. Review related guidelines
Shamsi, 2016 [60]	1. Focus groups; 2. Survey
Shive, 2006 [61]	1. Focus groups; 2. Interviews
Stead, 2017 [62]	1. Focus groups
Sun, 2007 [63]	1. Application of theory, and/or frameworks
Velema, 2018 [64]	1. Application of theory, and/or frameworks; 2. Interviews; 3. Focus groups; 4. Design specific to 4 Ps
Wilson, 2015 [65]	1. Focus groups; 2. Community stakeholder engagement; 3. Use communications firm
Wu, 2018 [66]	1. Interviews; 2. Focus groups; 3. Application of theory, and/or frameworks
Wyatt, 2022 [67]	1. Interviews; 2. Application of theory, and/or frameworks; 3. Design using the four Ps; 4: Co-creation
Pormehr-Yabandeh, 2023 [68]	1. Interviews

#### Categories of intervention strategies used to promote implementation

The two most common intervention categories were education (23/28, 82%) and providing (some subsidized, some at no cost) the actual goods that were central to performing the behaviour [e.g., providing car booster seats for free to those in need [41], initially subsidizing the fortified oil that was being encouraged [66], and provision of cooking pots [59] (22/28, 79%)]. The next most common categories included providing a service to participants [e.g., counselling (9/28, 32%)], training (6/28, 21%), and using local champions (5/28, 18%). See Table 5 for a complete list of all intervention categories used.

**Table 5:** Intervention Categories

**Intervention Category**	**Number (%) ** ***N*** **=28**
Education	23 (82%)
Product or subsidized product given	22 (79%)
Provision of a service (e.g., Consultation/counselling)	9 (32%)
Training	6 (21%)
Local champion	5 (18%)
Brand development	3 (11%)
Engaging spouses	3 (11%)
Anti-promotion (eg increase price of unhealthy food, making unhealthy food less available)	2 (7%)
Offering ‘other’ bonus services	2 (7%)
Peer support	2 (7%)
Policy	2 (7%)
Provision of money	1 (4%)
Advocacy	1 (4%)
Fines	1 (4%)
Emotional messaging	1 (4%)
Reminders	1 (4%)
Incorporation into routine paperwork	1 (4%)
Nudges	1 (4%)
Shaming	1 (4%)

Interventions could be coded to multiple categories

### Context, mechanism, outcome

Context varied considerably and is presented as raw data in the excel file in Additional File 2. We extracted mechanisms for all studies; thus, some degree of a reported mechanism existed for each study even if a formal theory was not itself referenced. Nineteen of the 28 studies (68%) contained mechanisms related to motivation and opportunity, six (21%) related to motivation, opportunity, and capability, one (4%) related solely to motivation, and one (4%) related solely to opportunity.

All 28 studies had an outcome focused on changing behaviour. Ten (36%) studies included knowledge outcomes, eight (29%) included attitude outcomes, and four (14%) included self-efficacy outcomes. Additional outcomes included anthropometric or physiological measures (3, 11%), intentions (2, 7%), and other (4, 14%).

See Additional File 2 for an Excel file of the raw extracted data from all studies including the five essential marketing criteria and study results.

### Team consensus activity

Our team meeting yielded the following seven commonly used approaches from social marketing that were felt to be important but underutilised in implementation science: Leverage emotions; Design for appeal; Consider what your audience values; Understand the price; Understand the place; Emphasize competitive advantage; and Use branding. Any of these approaches could be considered during the process of implementation strategy design (e.g., does emotion possibly play a role in our implementation goals). In addition, they could be considered for implementation strategies (e.g., use persuasive techniques to address the 'price' of behaviour change; position desired behaviours as superior to current behaviours; design strategies that are visually and conceptually appealing to the target audience) and mechanisms (e.g., measure changes in emotion levels as part of the strategy evaluation; determine if trust was achieved through branding). See Table 6 for a summary of these seven approaches including definitions/descriptions of concepts and examples from our review.

**Table 6:** Seven Approaches from Social Marketing Relevant to Implementation Science

**Approach**	**Definition/Defining Features**	**Social Marketing Example From Our Review**
**1. Leverage emotions**	Emotion is a complex reaction to a personally significant stimulus involving experiential, behavioral, and physiological elements [69]. This approach involves using strategically designed appeals in materials or messages that elicit an emotional reaction in order to enhance the audience’s engagement with the content.	A program emphasized the potential for social embarrassment of those with poor community hygiene practices in order to trigger a collective community emotional response [57].
**2. Design for appeal**	Appeal refers to framing or presenting the behaviour or intervention in a way that is attractive or desirable to the target audience [70]. This approach can involve designing strategies that are visually and conceptually appealing or fun to the target audience.	Demonstrations of healthy eating included preparation and distribution of 100% fruit juice smoothies to the crowd to highlight their tastiness and visual appeal [61].
**3. Consider your audience’s values **	Values are broad desirable goals that motivate people's action and serve as guiding principles in their lives [71]. This approach involves understanding the target audience’s core values through formative research and tailoring strategies and messaging to show how the behavior or its benefits align with these values.	Construction workers were encouraged to wear a helmet using a sticker reminding them of the importance of caring for themselves for their families (their highest value) [60].
**4. Understand the price**	Price is ‘what the target audience has to give up in exchange for adopting the behavior’ [70]. This approach considers the tangible, social, or emotional costs that may be involved in changing a specific behavior and designing strategies to reduce those barriers.	Videos of exercise classes were offered in order to increase exercise rates among those who felt lack of time was a barrier to in-person attendance [46].
**5. Understand the place**	Place considers where and when the opportunities are to engage in the behaviour as well as receive the intervention [70]. This approach considers how to design the audience’s physical and mental context to optimize strategies that support engagement in the target behaviour.	Friday night baseball games popular with families who drove there with their kids were considered a promising venue for promoting the use of booster seats for children [41].
**6. Emphasize competitive advantage**	Competition is what the audience is doing instead of the target behavior to achieve a specific goal — either a different behavior or inaction [70]. This approach considers how to position the target behavior as distinct and superior to the competing behaviors.	Pricing changes were undertaken to increase the cost of unhealthy food and decrease the cost of healthy food as a means to encourage healthy eating [64].
**7. Use branding**	A brand is a name, symbol, term, sign, design (or combination of these) that conveys multiple levels of meaning about a product: attributes, benefits (functional and emotional), values, culture, personality, and user image [72]. Using branding (often through multiple audience touchpoints) can influence how the audience thinks about and connects with the behavioral product, creating trust and loyalty.	A brand was created through a name, a mascot and symbols that were all culturally sensitive and meant to invoke trust in a project to encourage children to eat eggs [48].

## Discussion

This scoping review has highlighted social marketing approaches that could be applied to implementation science. The 28 included studies span over two decades. The geographical distribution of the included studies showcases a broad representation across 16 different countries, with most studies originating from the United States. This global diversity offers valuable insights into the applicability of social marketing interventions within various cultural, social, and economic contexts. The relatively extensive reporting of the use of theory, as well as the integration of formative work in social marketing intervention development, focuses attention on the value social marketing places on building interventions based on existing frameworks and tailoring strategies to target populations' specific needs and preferences. Social marketing interventions tend to be focused on health (or well-being) behaviour change (e.g., installing booster seats for children), but we have proposed here that the approaches can be usefully applied to a much broader range of implementation strategies, their design, and their mechanisms. The complexity of interventions reported, coupled with the emphasis on community engagement, highlights the multifaceted approaches employed to facilitate behaviour change. Our review outlined seven potential approaches from social marketing that could be considered promising avenues for future research and practice in implementation efforts.

Our review has shown that both overlap and difference exist between the fields of implementation science and social marketing. Areas of clear overlap include a focus on behaviour, the use of complex interventions, and an interest in some similar theories and frameworks (e.g., REAIM [33], Theoretical Domains Framework [73]). That said, differences were found. While formative work does occur in implementation science [74, 75], it is only recommended [76], unlike social marketing, where it is required. Similarly, while implementation science is often focused on behaviour change [76], this focus is not a required element of implementation science, as it is in social marketing.

Eighty-two percent of the studies used theory to inform their intervention design, a higher rate of theory use than what is typically seen in implementation science [11, 77, 78]. Although it is not certain whether these differences represent opportunities for advancing implementation efforts, our review has identified different theories that could help to broaden our perspective and that may usefully add to our toolkit of theories. One such theory is Exchange Theory, which proposes that people aim to optimize value for themselves by achieving the greatest benefit with the least cost (i.e., trade-offs) [25]. A second theory is the Consumer Information Processing Model, which draws attention to how consumers make decisions based on the information presented [29]. With 17 theories represented across the 28 social marketing studies, it is possible that the range of theoretical perspectives used in social marketing is consistent with the range of theoretical perspectives in implementation science [79] despite the application rate remaining low in implementation science.

Implementation science has a growing interest in explicating and reporting mechanisms (i.e., the underlying processes responsible for change) [80, 81] whereas social marketing appears more experienced with designing and reporting intervention mechanisms. We extracted mechanisms in all 28 social marketing studies. Although we did not assess whether these studies tested the mechanisms, we gained a reasonable understanding of the proposed processes for change. The mechanisms used in social marketing were predominantly focused on opportunity and motivation (the ‘O’ and ‘M’ in COM-B). Only 21% of the studies included mechanisms related to capability (the ‘C’ in COM-B). Finding the right mechanism is a function of the targeted barrier for behaviour change; however, it might be worthwhile to ask in which contexts capability (e.g., skill building) is more beneficial than motivation and opportunity. Studies exist in implementation science that describe active ingredients in implementation strategies using behaviour change techniques [82], and a review examined studies that tested mechanisms in implementation science [80], but certainly more work is needed in this area. The growing focus in implementation science on ensuring we design and report on mechanisms [83] is one important way to enhance our understanding of optimal mechanisms.

There were areas in which the overlap between social marketing and implementation science was not complete, which represented an opportunity for learning in terms of where emphasis might be placed. The idea of leveraging emotion is not absent from implementation science strategies or frameworks; some frameworks do encourage an examination of emotion (e.g. one of the 14 domains in the Theoretical Domains Framework [73] is emotion and Dual Process models [84] include considering the impulsive/emotional aspect of behaviour change alongside the cognitive aspects), but in social marketing, emotion plays a more central role including frameworks that solely explain emotion [85]. Likewise with appeal. When an implementation scientist focuses on utility or usability of an implementation strategy [86, 87] they invoke concepts related to appeal, but are not necessarily focused on appeal or enjoyment. Last, there may be some differences in the emphasis placed on understanding values. The literature on shared decision-making places significant focus on patient values [88] as does the growing interest in patient engagement [89, 90]. However, it remains unclear the degree to which understanding the audience’s values is central to implementation science. For example, implementation scientists generally assume that their audience values evidence-based practice [91], but the degree of this value is rarely measured or incorporated into implementation strategy design, the strategies themselves, or the mechanisms that are responsible for change. If considering what our audience values is important then more efforts to examine these values might be warranted.

The concept of *Price* in social marketing might represent one of the most important approaches to consider. Specifically, a value-based pricing approach should be considered, where the value of the offering (behaviour change) for the customer is considered when assessing their willingness to engage in the desired behaviour. Incorporating this concept into implementation science has the potential to enrich implementation strategy design by considering the broader costs associated with behaviour change. Indeed, there is a growing call in economic evaluation in implementation science to use mixed methods (e.g., focus groups. ethnography) alongside traditional quantitative economic evaluations to better understand the range of indirect and intangible costs to implementation efforts [92–94]. Understanding what individuals must sacrifice could inform tailored strategies to address perceived barriers and enhance participant engagement. By framing behaviour change as a transactional process, implementation science could employ ‘pricing strategies’ to influence decision-making more effectively. The concept of price also could have implications for framing de-adoption. De-adoption frameworks, like the one proposed by Niven and colleagues [95], emphasize the importance of stakeholder engagement and robust barriers assessment that includes historical, political, social, and economic factors but does not necessarily consider the price to an individual of de-adoption. Interventions likely to promote de-adoption include policy changes and changes to funding models [95]; however, these approaches do not consider how to lower the price or make it easier for people to change.

The seven social marketing approaches we have identified offer much potential to be translated into testable hypotheses within implementation science. For instance, hypotheses could be formulated to investigate the effectiveness of leveraging emotions in implementation strategies, the influence of knowing what might appeal to the target group, or the role of competition in promoting healthcare provider behaviour change within healthcare settings. Additionally, hypotheses could explore the impact of understanding audience values on the implementation strategy acceptability and engagement, the effectiveness of considering pricing strategies (i.e., the costs associated with the behaviour change) when designing an implementation strategy, and the role of branding in enhancing adoption and sustainability of an evidence-based intervention. There could be benefits to integrating combinations of the seven social marketing approaches we have identified. For example, by leveraging emotion, designing implementation strategies for appeal, and considering what the audience values, implementation scientists could create strategies that resonate more deeply with their audience. This could lead to increased engagement, motivation, and ultimately, more successful implementation outcomes. Alternatively, incorporating social marketing approaches, such as understanding the target audience's values and cultural norms, could enhance strategy relevance and cultural competence. Aligning with people’s cultures and values may help overcome barriers related to cultural differences and ensure that strategies are respectful and inclusive of diverse populations. Future research should encourage collaboration between implementation scientists and social marketers.

Indeed, other related fields such as behavioural economics are being examined for integration into implementation science [96]. All of these novel but overlapping areas could facilitate the exchange of knowledge, expertise, and best practices.

This study is not without limitations. Despite our team comprising five implementation scientists, we had limited representation with only one marketing scientist, one social marketing practitioner, and one individual bridging across disciplines. The team would have benefited from additional expertise in marketing science to enhance our collective capabilities. All the studies we found were in health, which might have limited the degree to which we found novel strategies. We used the stringent inclusion criteria of the study having to test an intervention with a control as we wanted to ensure robust intervention descriptions, and it was a very new field for the majority of our team. Now that we understand this field better, and know that controlled studies are more uncommon, future searches could be conducted that include other study designs. We used a recommended conceptualization of essential social marketing criteria [7] to guide our extraction but found the variety in the extracted data a challenge to meaningfully synthesize. Perhaps as our understanding of social marketing increases, synthesizing such variety could become more achievable. As this was our first attempt to learn from the field of social marketing, our seven suggested social marketing approaches are more broad than practical, and additional work is required to fully appreciate how they might be meaningfully applied, despite the examples we provided. We did this in part to avoid assumptions or quick applications that might not properly align to social marketing. Additional work is needed in this area with the guidance of social marketing expertise. To this end, an integrative review of the two literatures might be a useful next step in our understanding of social marketing and would have the added benefit of bi-directional understanding and value. We are aware that we created a set of broad intervention categories/labels inductively to describe these social marketing interventions when implementation science offers a proliferation of taxonomies and strategy lists [97, 98]. Using one of the implementation science frameworks might have allowed for a more robust coding process with terms familiar to implementation scientists. However, we were reluctant to use an implementation science lens to describe the social marketing interventions for fear that doing so might prevent us from seeing novel trends. Future work should determine if and to what degree social marketing interventions are aligned to taxonomies of implementation strategies. This would help determine what might be unique to each field and point to potential synergy between these fields. We did not use the Template for Intervention Description and Replication (TIDieR) Checklist [99] to assess reporting in these studies as we did not ask a question about completeness of reporting, but this too is a worthwhile endeavour in future work. The consensus discussions to arrive at our seven social marketing approaches was done only within the research team. Hence, we did not use the term ‘consultation exercise’ in this scoping review [100, 101]. We deemed it too labour intensive to ask additional knowledge users to read through the complete extraction table but certainly a fulsome consultation exercise with researchers in implementation science or other audiences outside of the team might have yielded different approaches. A broader consultation would also mitigate the possibility that seniority or differing expertise on our research team impacted engagement or sharing of ideas.

## Conclusion

Social marketing and implementation science share common goals of promoting behaviour change and improving health outcomes, yet they approach these objectives from distinct perspectives. Despite differences, there is significant potential for synergy between the two fields, as they offer complementary perspectives and methodologies for designing, implementing, and evaluating interventions aimed at improving health. Incorporating social marketing concepts into implementation science has the potential to enhance the effectiveness, relevance, and sustainability of behaviour change interventions. By leveraging approaches from social marketing, implementation scientists could develop more engaging, culturally sensitive, and impactful interventions, ultimately leading to positive behaviour change outcomes and improvements in health.

## Supplementary Information

Additional file 1. full set of search strategies.

Additional file 2. excel document of all extracted data.

## Data Availability

All data generated or analysed during this study are included in this published article [and its supplementary information files].

## Abbreviations

PRISMA-ScR: Preferred Reporting Items for Systematic Reviews and Meta-Analyses Extension for Scoping Reviews
PRESS: Peer-Review of Electronic Search Strategies
COM-B: Capability, Opportunity, Motivation, Behaviour.
CMO: Context, mechanism, outcome
JBI: Joanna Briggs Institute
RE-AIM: Reach, Effectiveness, Adoption, Implementation, and Maintenance
