# Supplementary material for: Applications of social marketing for implementation science: a scoping review
Source: Implement Sci. 2025 Nov 29;20:52. doi: 10.1186/s13012-025-01458-z (PMC12670846; doi:10.1186/s13012-025-01458-z)
Supplement: Supplementary file 1 — Additional file 1. full set of search strategies. [file 13012_2025_1458_MOESM1_ESM.docx]

**Supplemental Material A**

**Search updated on January 30, 2024. Total: 390 (After removing duplicates n=287)**

**OVID=207**

Embase Classic+Embase <1947 to 2024 January 30>

APA PsycInfo <1806 to February Week 3 2024>

Journals@Ovid Full Text < January 30, 2024>

Ovid MEDLINE(R) ALL <1946 to January 30, 2024>

| 1 | Social marketing.ti,kf. |
| --- | --- |
| 2 | marketing.ti,kf. |
| 3 | ((Social marketing or marketing) adj5 behavior*).ti,ab. |
| 4 | ((Social marketing or marketing) adj5 behavior change*).ti,ab. |
| 5 | ((Social marketing or marketing) adj5 campaign*).ti,ab. |
| 6 | ((Social marketing or marketing) adj5 communication*).ti,ab. |
| 7 | ((Social marketing or marketing) adj5 (customer* or consumer)).ti,ab. |
| 8 | ((Social marketing or marketing) adj5 promotion*).ti,ab. |
| 9 | ((Social marketing or marketing) adj5 health*).ti,ab. |
| 10 | ((Social marketing or marketing) adj5 knowledge translation*).ti,ab. |
| 11 | ((social marketing or marketing) adj5 (intervention* or strategy* or effect* or effectiveness or impact* or success* or improve* or failure*)).ti,ab. |
| 12 | randomized controlled trial.pt. |
| 13 | controlled clinical trial.pt. |
| 14 | randomized.ab. |
| 15 | randomly.ab. |
| 16 | trial.ab. |
| 17 | Quasi experimental.ti,ab. |
| 18 | 1 or 2 or 3 or 4 or 5 or 6 or 7 or 8 or 9 or 10 or 11 |
| 19 | 12 or 13 or 14 or 15 or 16 or 17 |
| 20 | 18 and 19 |
| 21 | limit 20 to english language |
| 22 | limit 21 to human |
| 23 | limit 22 to humans |
| 24 | limit 23 to yr="2023 -Current" |
| 25 | limit 24 to english language |

**Pubmed:19**

**("social marketing"[Title] OR "marketing"[Title])) OR ((("social marketing"[Title] OR "marketing"[Title]) and "promotion*"[Title]) ) OR (((("social marketing"[Title] OR "marketing"[Title]) and "behavior*"[Title]))) OR (((("social marketing"[Title] OR "marketing"[Title]) and "behavior change*"[Title]))) OR (((("social marketing"[Title] OR "marketing"[Title]) and "campaign*"[Title]))) OR (((("social marketing"[Title] OR "marketing"[Title]) and "communication*"[Title]) ))) OR (((("social marketing"[Title] OR "marketing"[Title]) and "consumer*"[Title]) ))) OR ((("social marketing"[Title] OR "marketing"[Title]) and "intervention*"[Title]) ))) OR (((("social marketing"[Title] OR "marketing"[Title]) and "trial*"[Title]) ))) OR (((("social marketing"[Title] OR "marketing"[Title]) and "strateg*"[Title]) ))) OR (((("social marketing"[Title] OR "marketing"[Title]) and "effect*"[Title]) ))) OR (((("social marketing"[Title] OR "marketing"[Title]) and "impact*"[Title]) ) OR (((("social marketing"[Title] OR "marketing"[Title]) and "success*"[Title]) ) OR (((("social marketing"[Title] OR "marketing"[Title]) and "failure*"[Title]) ) AND ("randomized controlled trial"[pt]OR "quasi-experimental" [Title/Abstract] OR "randomized" [Title/Abstract] OR "randomized control"[Title/Abstract] OR "controlled trial"[Title/Abstract]) Filters: English, from 2023 – 2024**

**Scopus:79**

( TITLE ( "social marketing" OR "marketing" ) OR TITLE-ABS ( ( "social marketing" OR "marketing" ) W/5 promotion* ) OR TITLE-ABS ( ( "social marketing" OR "marketing" ) W/5 behavior* ) OR TITLE-ABS ( ( "social marketing" OR "marketing" ) W/5 behavior AND change* ) OR TITLE-ABS ( ( "social marketing" OR "marketing" ) W/5 health ) OR TITLE-ABS ( ( "social marketing" OR "marketing" ) W/5 campaign* ) OR TITLE-ABS ( ( "social marketing" OR "marketing" ) W/5 communication* ) OR TITLE-ABS ( ( "social marketing" OR "marketing" ) W/5 theor* ) OR TITLE-ABS ( ( "social marketing" OR "marketing" ) W/5 intervention* ) OR TITLE-ABS ( ( "social marketing" OR "marketing" ) W/5 trial* ) OR ( ( "social marketing" OR "marketing" ) W/5 strateg* ) OR TITLE-ABS ( ( "social marketing" OR "marketing" ) W/5 ( "effect*" OR "effectiveness" OR "impact*" OR "success" OR "improve*" OR "failure" ) ) AND ( TITLE-ABS ( "quasi experimental" OR "randomized controlled trial" OR " randomized" OR " controlled trial" OR "randomized control") ) ) AND NOT ( infant OR children OR adolescents ) AND ( EXCLUDE ( LANGUAGE,"English" ) ) AND ( LIMIT-TO ( EXACTKEYWORD,"Human" ) OR LIMIT-TO ( EXACTKEYWORD,"Controlled Study" ) OR LIMIT-TO ( EXACTKEYWORD,"Randomized Controlled Trial" ) OR LIMIT-TO ( EXACTKEYWORD,"Humans" ) OR LIMIT-TO ( EXACTKEYWORD,"Controlled Clinical Trial" ) OR LIMIT-TO ( EXACTKEYWORD,"Randomized Controlled Trial (topic)" ) ) AND ( LIMIT-TO ( PUBYEAR,2023-2024) )

**CINHAL:42**

( TI ( "social marketing" OR "marketing" ) OR AB ( ( "social marketing" OR "marketing" ) N5 promotion* ) OR AB ( ( "social marketing" OR "marketing" ) N5 behavior* ) OR AB ( ( "social marketing" OR "marketing" ) N5 behavior AND change* ) OR AB ( ( "social marketing" OR "marketing" ) N5 health ) OR AB ( ( "social marketing" OR "marketing" ) N5 campaign* ) OR AB ( ( "social marketing" OR "marketing" ) N5 communication* ) OR AB ( "social marketing" OR "marketing" ) N5 customer* ) OR AB ( "social marketing" OR "marketing" ) N5 consumer* ) OR AB ( ( "social marketing" OR "marketing" ) N5 theor* ) OR AB ( ( "social marketing" OR "marketing" ) N5 intervention* ) OR AB ( ( "social marketing" OR "marketing" ) N5 trial* ) OR ( ( "social marketing" OR "marketing" ) N5 strateg* ) OR AB ( ( "social marketing" OR "marketing" ) N5 ( "effect*" OR "effectiveness" OR "impact*" OR "success" OR "improve*" OR "failure" ) ) AND ( AB ( "quasi experiment*" OR "randomized controlled trial*" OR “randomized control” OR “randomized”) ) ) **Limiters** - Published Date: 20230101-20241231; English Language; Human; Age Groups: All Adult; Language: English

**Expanders** - Apply equivalent subjects

**Search modes** - Boolean/Phrase

**Cochrane: 26**

| Search Name: |
| --- |
| Date Run: 30/01/2024 |
| ID Search Hits |
| #1 ("social marketing" or "marketing"):ti |
| #2 (("Social marketing" or "marketing") NEAR/5 behavior*):ti |
| #3 (("Social marketing" or "marketing") NEAR/5 behavior change*):ti |
| #4 ((Social marketing or marketing) NEAR/5 campaign*):ti |
| #5 (("Social marketing" or "marketing") NEAR/5 (customer* or consumer*)):ti |
| #6 (("Social marketing" or "marketing") NEAR/5 promotion*):ti |
| #7 (("Social marketing" or "marketing") NEAR/5 health*):ti |
| #8 (("Social marketing" or "marketing") NEAR/5 knowledge translation*):ti |
| #9 (("Social marketing" or "marketing") NEAR/5 behavior change*):ti |
| #10 (("social marketing" or "marketing") NEAR/5 ("intervention*" or "strategy*" or "effect*" or "effectiveness" or "impact*" or "success*" or "improve*" or "failure*")):ti |
| #11 #1 or #2 or #4 or #5 or #6 or #7 or #8 or #9 or #10 |
| with Publication Year from 2023 to 2024, with Cochrane Library publication date from Jan 2023 to Mar 2024, in Trials |

ERIC: 17

(TI ("social marketing" OR "marketing") OR AB (("social marketing" OR "marketing") NEAR/5 promotion*) OR AB (("social marketing" OR "marketing") NEAR/5 behavior*) OR AB (("social marketing" OR "marketing") NEAR/5 behavior AND change*) OR AB (("social marketing" OR "marketing") NEAR/5 health) OR AB (("social marketing" OR "marketing") NEAR/5 campaign*) OR AB (("social marketing" OR "marketing") NEAR/5 communication*) OR AB (("social marketing" OR "marketing") NEAR/5 theor*) OR AB (("social marketing" OR "marketing") NEAR/5 intervention*) OR AB (("social marketing" OR "marketing") NEAR/5 trial*) OR (("social marketing" OR "marketing") NEAR/5 strateg*) OR AB (("social marketing" OR "marketing") NEAR/5 ("effect*" OR "effectiveness" OR "impact*" OR "success" OR "improve*" OR "failure")) AND (AB (("quasi experiment" OR "quasi experimental" OR "quasi experiments") OR "randomized controlled trial*" OR "randomized" OR "controlled trail")))Additional limits - Date: From January 01 2023 to December 31 2024

CDR, NICE, Web of science: No new evidence!
